# Analysis of brain connectivity during nitrous oxide sedation using graph theory

**DOI:** 10.1038/s41598-020-59264-0

**Published:** 2020-02-11

**Authors:** Ji-Min Lee, Pil-Jong Kim, Hong-Gee Kim, Hong-Keun Hyun, Young Jae Kim, Jung-Wook Kim, Teo Jeon Shin

**Affiliations:** 1 Department of Pediatric Dentistry and Dental Research Institute, School of Dentistry, Seoul National University, Seoul, Republic of Korea; 2 Biomedical Knowledge Engineering Laboratory, School of Dentistry, Seoul National University, Seoul, Republic of Korea

**Keywords:** Consciousness, Network models

## Abstract

Nitrous oxide, the least potent inhalation anesthetic, is widely used for conscious sedation. Recently, it has been reported that the occurrence of anesthetic-induced loss of consciousness decreases the interconnection between brain regions, resulting in brain network changes. However, few studies have investigated these changes in conscious sedation using nitrous oxide. Therefore, the present study aimed to use graph theory to analyze changes in brain networks during nitrous oxide sedation. Participants were 20 healthy volunteers (10 men and 10 women, 20–40 years old) with no history of systemic disease. We acquired electroencephalogram (EEG) recordings of 32 channels during baseline, nitrous oxide inhalation sedation, and recovery. EEG epochs from the baseline and the sedation state (50% nitrous oxide) were extracted and analyzed with the network connection parameters of graph theory. Analysis of 1/f dynamics, revealed a steeper slope while in the sedation state than during the baseline. Network connectivity parameters showed significant differences between the baseline and sedation state, in delta, alpha1, alpha2, and beta2 frequency bands. The most pronounced differences in functional distance during nitrous oxide sedation were observed in the alpha1 and alpha2 frequency bands. Change in 1/f dynamics indicates that changes in brain network systems occur during nitrous oxide administration. Changes in network parameters imply that nitrous oxide interferes with the efficiency of information integration in the frequency bands important for cognitive processes and attention tasks. Alteration of brain network during nitrous oxide administration may be associated to the sedative mechanism of nitrous oxide.

## Introduction

Behavior management required for children with severe anxiety and fear can lead to difficulties in obtaining high quality dental care. In adults, pain or anxiety may cause reluctance to receiving dental treatment. In particular, in patients with a history of syncope due to severe stress, dentists should attempt to minimize stress, ensure that patients are comfortable with the treatment, and consider the use of sedation during dental treatment.

Nitrous oxide is a sedative, widely used in medicine and dentistry^1^ due to its sedative and analgesic effects, fast onset and recovery, and lack of serious side effects. Sedation refers to the intermediate state between consciousness and total loss of consciousness, in which consciousness is suppressed. Nitrous oxide is the least potent inhalation anesthetic, and induces a minimally conscious sedation state allowing patients to respond appropriately to physical stimuli or verbal commands^2^.

The brain is a complex network, and recently using the graph theoretical approach, the structural and functional network of the brain has been shown to have similar properties as those of other complex network systems^3^. Graph theory is a mathematical field that attempts to understand and analyze social phenomena, nature, and network structure, by simplifying them to graphs, defined as a set of nodes (also called “vertices”) connected by edges (also called “lines”). Despite differences in the details of each system element, the complex network is known to have similar macroscopic behavior^4^. Using graph theory, the application is not influenced by the kind of nodes and edges. Therefore, the same network analysis can be applied^5^ and the efficiency of information exchange within a network can be mathematically explained.

From this point of view, the brain network is a huge complex network system that consists of nodes represented by neurological elements such as neurons or brain regions, and connecting edges represented by axonal projections or synapses. One characteristic of this complex network is small world topology^4,6^, which features efficient local and long distance connections^7^. Previous studies have reported loss of small world topology^8–10^ in patients with neurological disease.

In previous reports, it can be inferred that cognition changes, widely observed for patients with neurological disorders may be related to the disorganization of brain network. In agreement with our hypothesis, brain network and functional connectivity are altered by anesthetic-induced loss of consciousness^11,12^. However, little studies have been done on how brain networks change due to subtle changes in consciousness levels although nitrous oxide is widely used in dentistry for conscious sedation. For investigating sedation mechanism of nitrous oxide, many reports have been made about the effect of nitrous oxide on EEG activity. Rampil *et al*. reported an increase in high beta (40–50 Hz) activity^13^, whereas Yamamura *et al*. reported an increase in fast oscillatory activity with a decrease in alpha activity at 50% nitrous oxide concentration^14^. In contrast, Liley and Foster reported a decrease in delta power at 20, 40% concentration of nitrous oxide sedation in 2011 and 2013 studies^15,16^. However, mechanism of brain network changes during nitrous oxide has not been studied. And changes in functional connectivity during nitrous oxide sedation are yet to be demonstrated from the global brain network perspective. Understanding the state of the brain network during sedation state can help to understand the loss of consciousness and the mechanism of recovery. Therefore, the aim of this study was to use graph theory to analyze brain network changes during nitrous oxide sedation.

## Results

### 1/f dynamics

**Figure 1:**
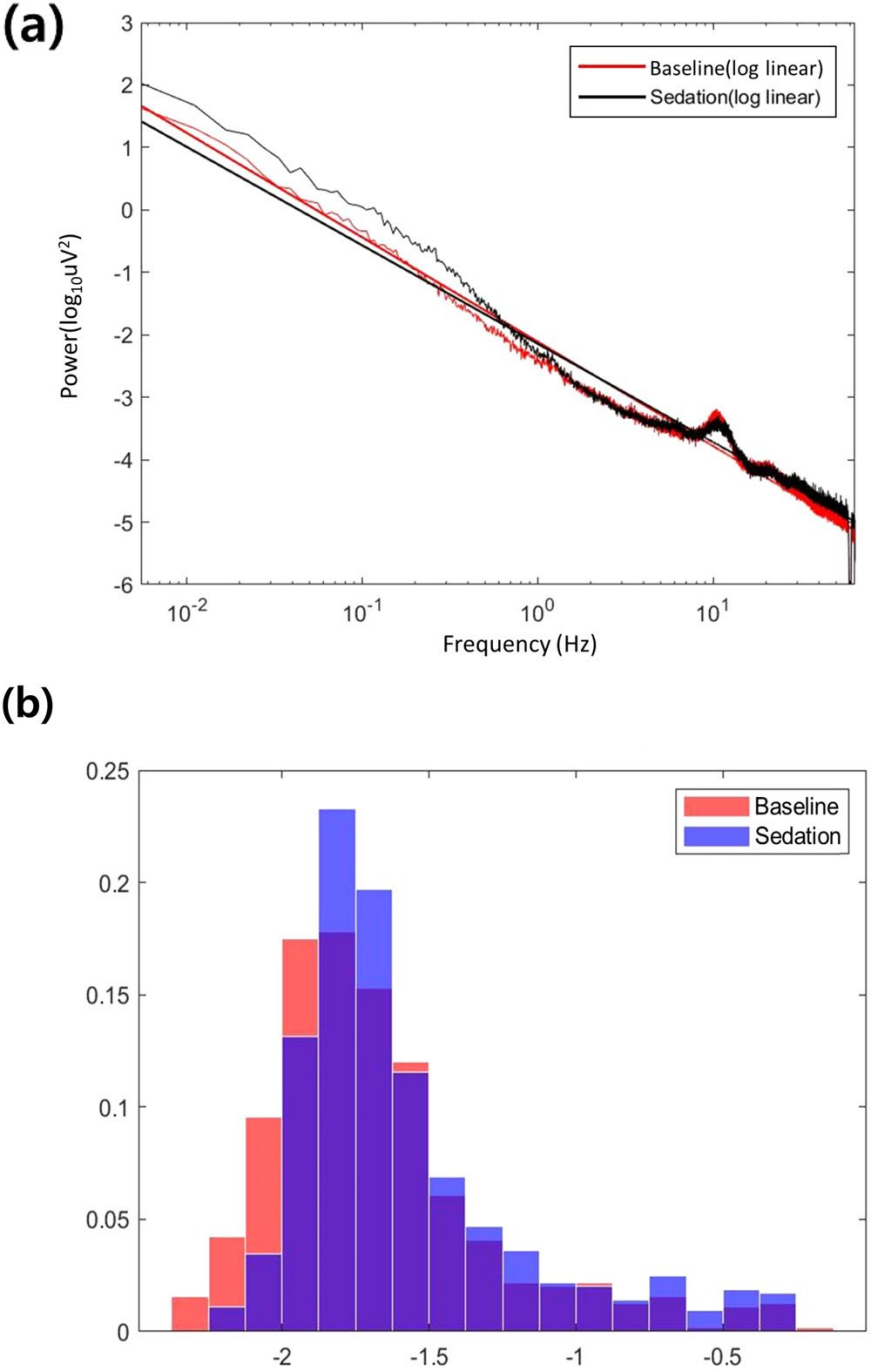
(**a**) The representatives of log(1/f) dynamics for the baseline (red) and the sedation state (black). (**b**) The slope histogram of all log(1/f) dynamics in baseline (red) and sedation status (blue). X axis represents the coefficients of slopes and y axis represents its proportions, respectively.

The slope of the baseline was significantly steeper than that of the sedation state (Fig. 1a,b *p* < 0.001), indicating of the power spectra of random noise during nitrous oxide sedation. This suggests that nitrous oxide sedation favors a shift to a more-random network (Fig. 1).

### Network connectivity analysis

**Figure 2:**
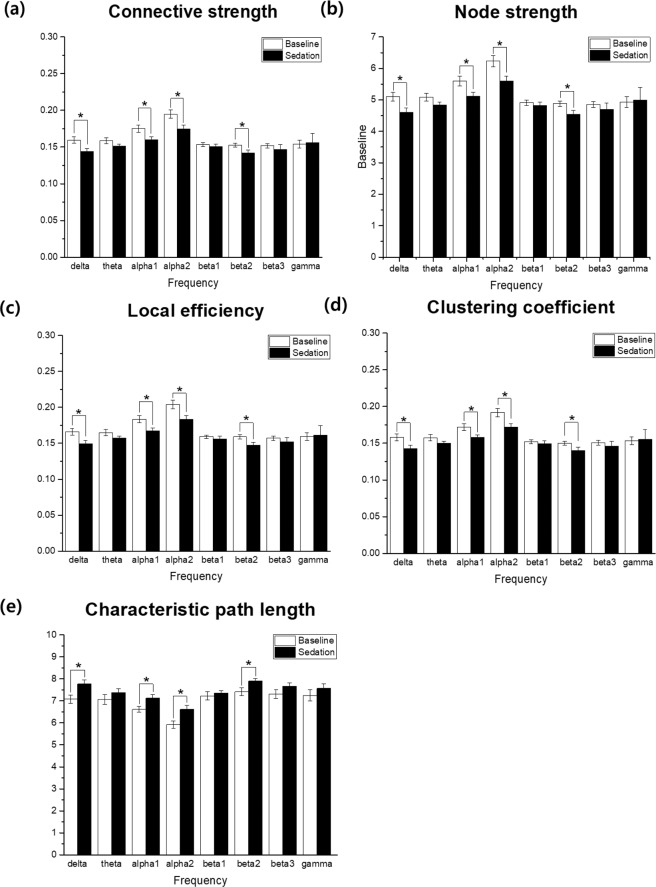
Network connectivity parameters of the baseline (white) and the sedation state (black) frequency bands. (**a**) Connectivity strength, (**b**) Node strength, (**c**) Local efficiency, (**d**) Clustering coefficient, and (**e**) Characteristic path length (*p < 0.05). The error bars indicate standard error.

We found an overall difference between the baseline and the sedation state, in the mean values of connectivity strength, node strength, clustering coefficient, local efficiency, and characteristic path length. Specifically, during the sedation state, delta, alpha1, alpha2, and beta2 frequency bands had significantly lower connective strength, node strength, clustering coefficient, and local efficiency, than during the baseline. Characteristic path length was significantly higher in the sedation state compared to the baseline (Fig. 2). The correlation coefficients between path length and other parameters were very high. The specific correlation values were as follows: Connective strength: −0.947, Node strength: −0.947, Clustering coefficient: −0.946, Local efficiency: −0.948. This suggests that increased path length is strongly correlated to decreased connective strength, node strength, clustering coefficient, and local efficiency. No differences in any network parameters were observed between males and females (data not shown). For all network parameters, the most prominent differences between the baseline and sedation state were seen in the alpha 1 and 2 frequency bands.

Differences in brain network connectivity between the baseline and sedation state are summarized in Table 1.

**Table 1:** Mean value and standard deviation of network connectivity parameters between the control and sedation state (*p < 0.05).

Parameter	Frequency	state	Mean	SD	*p* Value
Connective strength	delta	Baseline	0.160	0.02	0.007^*^
	delta	Sedation	0.144	0.02	
	theta	Baseline	0.159	0.018	0.185
	theta	Sedation	0.151	0.013	
	alpha1	Baseline	0.175	0.022	0.007^*^
	alpha1	Sedation	0.160	0.017	
	alpha2	Baseline	0.195	0.026	0.002^*^
	alpha2	Sedation	0.175	0.023	
	beta1	Baseline	0.153	0.012	0.444
	beta1	Sedation	0.151	0.016	
	beta2	Baseline	0.152	0.012	0.046^*^
	beta2	Sedation	0.142	0.018	
	beta3	Baseline	0.152	0.014	0.240
	beta3	Sedation	0.147	0.029	
	gamma	Baseline	0.154	0.024	0.255
	gamma	Sedation	0.156	0.059	
Node strength	delta	Baseline	5.105	0.653	0.007^*^
	delta	Sedation	4.602	0.646	
	theta	Baseline	5.085	0.59	0.185
	theta	Sedation	4.841	0.413	
	alpha1	Baseline	5.602	0.717	0.007^*^
	alpha1	Sedation	5.121	0.560	
	alpha2	Baseline	6.230	0.821	0.002^*^
	alpha2	Sedation	5.597	0.731	
	beta1	Baseline	4.909	0.378	0.444
	beta1	Sedation	4.822	0.520	
	beta2	Baseline	4.880	0.391	0.046^*^
	beta2	Sedation	4.537	0.576	
	beta3	Baseline	4.856	0.436	0.240
	beta3	Sedation	4.695	0.935	
	gamma	Baseline	4.926	0.772	0.255
	gamma	Sedation	4.989	1.893	
Local efficiency	delta	Baseline	0.166	0.021	0.005^*^
	delta	Sedation	0.149	0.021	
	theta	Baseline	0.165	0.019	0.162
	theta	Sedation	0.157	0.013	
	alpha1	Baseline	0.184	0.024	0.007^*^
	alpha1	Sedation	0.167	0.019	
	alpha2	Baseline	0.204	0.027	0.003^*^
	alpha2	Sedation	0.184	0.024	
	beta1	Baseline	0.159	0.012	0.444
	beta1	Sedation	0.156	0.017	
	beta2	Baseline	0.159	0.013	0.042^*^
	beta2	Sedation	0.147	0.019	
	beta3	Baseline	0.157	0.014	0.225
	beta3	Sedation	0.152	0.03	
	gamma	Baseline	0.159	0.025	0.255
	gamma	Sedation	0.161	0.061	
Clustering coefficient	delta	Baseline	0.158	0.022	0.009^*^
	delta	Sedation	0.143	0.021	
	theta	Baseline	0.158	0.020	0.211
	theta	Sedation	0.150	0.014	
	alpha1	Baseline	0.172	0.022	0.008^*^
	alpha1	Sedation	0.158	0.017	
	alpha2	Baseline	0.192	0.026	0.002^*^
	alpha2	Sedation	0.172	0.022	
	beta1	Baseline	0.152	0.012	0.538
	beta1	Sedation	0.150	0.017	
	beta2	Baseline	0.150	0.012	0.050^*^
	beta2	Sedation	0.140	0.018	
	beta3	Baseline	0.151	0.014	0.240
	beta3	Sedation	0.146	0.030	
	gamma	Baseline	0.153	0.024	0.255
Characteristic path length	delta	Baseline	0.155	0.061	0.013^*^
	delta	Sedation	7.078	0.847	
	theta	Baseline	7.768	1.059	0.255
	theta	Sedation	7.066	0.828	
	alpha1	Baseline	7.376	0.624	0.008^*^
	alpha1	Sedation	6.617	0.747	
	alpha2	Baseline	7.124	0.742	<0.001^*^
	alpha2	Sedation	5.926	0.840	
	beta1	Baseline	6.619	0.842	0.49
	beta1	Sedation	7.229	0.522	
	beta2	Baseline	7.361	0.803	0.046^*^
	beta2	Sedation	7.419	0.552	
	beta3	Baseline	7.899	0.925	0.225
	beta3	Sedation	7.318	0.657	
	gamma	Baseline	7.675	1.142	0.185
	gamma	Sedation	7.252	0.929	

### Functional distance changes

**Figure 3:**
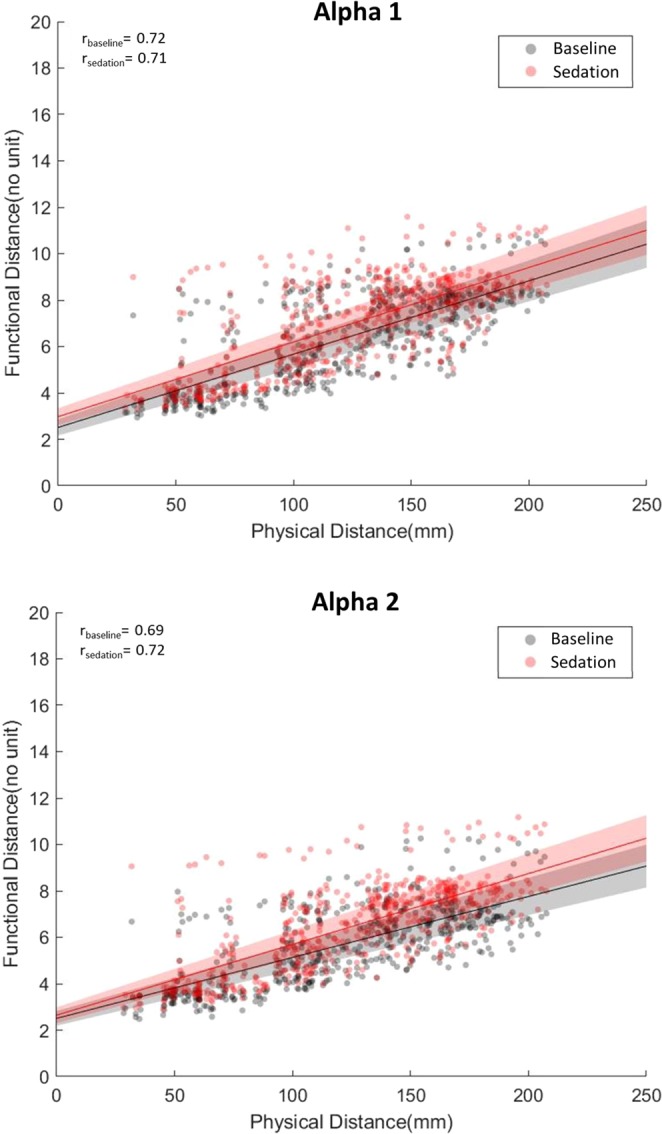
Scatter plot between functional and physical distance of pair-wise areas in the baseline (gray) or sedation (red) state, for alpha1 (top) and alpha2 (bottom) frequency bands. The red and black lines indicate the slope of the correlation in sedated and baseline states, respectively. The red and gray shaded areas indicate confidence bands for correlation coefficients in sedated and baseline states, respectively. R_baseline_ and R_sedation_ indicates the coefficients of correlation in sedated and baseline states, respectively.

The functional distance (in regard to each same physical distance pair), was longer in the sedation state than the baseline for the alpha1 and alpha2 frequency bands (Fig. 3) in which binominal p-values were < 0.001. It suggests that information efficiency was impaired during nitrous oxide sedation considering that the functional distance is defined as the shortest path length of the network. However, the effects of nitrous oxide on changes in correlation coefficients were not consistent (Fig. 3).

## Discussion

In this study, we used graph theory to analyze EEG data recorded during nitrous oxide sedation. We confirmed that in the sedation state, brain network properties differ from those in the baseline. To the best our knowledge, this is the first study to use graph theory to investigate brain network changes associated with nitrous oxide sedation.

Compared to the baseline, changes in the pattern of 1/f dynamics were observed during the sedation state. Electrophysiologically, power spectrum density follows an inverse relationship between logarithmical power - frequency domain^17,18^. Recent study have shown that excitatory and inhibitory balance determine 1/f dynamics on neural system^19^. In this study, we found that the slope of power law relationship indicative of orderness of network was changed during nitrous oxide sedation, suggesting that nitrous oxide sedation changed into characteristics of brain networks with random noise dominance. These changes have been observed in many neurological diseases, such as schizophrenia^20^, autism^21^, and anxiety^22^. From the perspective of network system, Pink noise is in equilibrium between order and randomness^23^, which allows network system to respond to external stimuli in an efficient way, and then returns to its original state^24^. This implies a shift to more randomness, which can indicate less coordinated signal organization or more random processing of information^21,25^. Indeed, as sedation depth increases, most patients experience amnesia and show slurred responses to verbal commands during sedation in most cases. This may be due to a change in network characteristics from pink to random noise that hampers the efficiency of responses to external stimuli. The goal of current research was to determine whether sedation is a new property of a changed network topology, which could be recognized by certain oscillatory pattern and connectivity signatures differing from that of normal subjects. It could be found that complex dynamics can be generated by systems fulfilling the following two requirements, the presence of noise and a “small-world” topology under general conditions^26^. By definition, complex adaptive systems typically generate complex output signals that have a 1/f decay of the power spectra^27^. The 1/f change in sedated patient suggests more randomness high frequency oscillatory activity in the brain network. The 1/f pink noise behavior in the network has been interpreted within the theory of self-organized criticality^28^. In contrast, in sedative patients a shift-to-randomness may imply less coordinated signal organization at the local level of possibly neural circuits. A 1/f-like power spectrum is indicative of arrhythmic brain activity that does not contain a predominant temporal scale (hence, “scale-free”)^29^. Therefore, 1/f dynamics could explain the aspects of noise presence in brain connectivity as complex adaptive systems

In addition to 1/f dynamics, parameters such as connective strength, node strength, clustering coefficient, local efficiency, and characteristic path length, are commonly used as indicators of network connectivity to decipher the features of network system^4,6^. Small world networks feature small path lengths and high clustering coefficients^7^. In this study, while in the sedation state, network parameters including the clustering coefficient and local efficiency were decreased, and the characteristic path length was increased, in several frequency bands, suggesting a change in small world network topology. Information integration theory suggests that certain cortical areas can distinguish and recognize varied information, and information is integrated by the connections between different areas and within these areas^30,31^. The efficiency of information transmission with neighboring nodes or directly connected nodes can be measured using local efficiency, and the efficiency of information transmission in the overall system can be measured through global efficiency^32^. Local efficiency and clustering coefficients can also be used to evaluate information segregation, while characteristic path length and global efficiency can measure information integration^32,33^. This study shows that nitrous oxide degrades both information integration and segregation. It can also be seen that nitrous oxide reduces both the transmission of information between and within the region of the brain network, resulting in a sedation. It is surprising that while in the sedation state, a reduction in the efficiency of information processing and distribution is observed, indicated by increased path length resulting from decreased local efficiency although nitrous oxide sedation is not quite different from the awake state phenomenonically. This indicates that nitrous oxide sedation is in a different state from awake state as evidenced by changes in network parameters and 1/f pattern. Studies using graph theory to analyze the loss of consciousness induced by propofol^34–36^, isoflurane^37^, and dexmedetomidne^12^, have revealed changes in network parameters, suggesting that anesthetics alter the brain functions associated with information transferring processes within the brain network. Consistent with previous research, nitrous oxide interferes with the efficiency of information integration, leading to the transition from the baseline to sedative state. Contradictory to a previous study in which propofol was reported to increase the clustering coefficient during general anesthesia^34,36^, we observed a decrease in the clustering coefficient of sedation. This may be due to differences in the drug used (propofol vs nitrous oxide), and the level of unconsciousness. Specifically, changes in brain network properties during nitrous oxide sedation may be caused by different mechanisms than that of the unconscious state induced by propofol. The decrease of clustering coefficient indicates the breakdown of subnetworks, the hallmark of small world network. In addition, changes in connective and node strengths indicate that nitrous oxide changes the quality of information processing in the brain network, and decreases the connection strength between nodes while in the sedation state^30,36^. Therefore, nitrous oxide may break down the network hub, densely connected nodes, thereby decreasing the efficiency of information processing to external stimuli. It can be seen that changes in consciousness associated with nitrous oxide sedation are related to changes in the quality of information processing and the change in link strength between regions.

In this experiment, characteristic path length had highly negative correlation with connective strength, node strength, clustering coefficient and local efficiency. The characteristic path length and local efficiency are measures of connectedness, estimating how easily information can be integrated into the network^38^. Compared to characteristic path length, local efficiency is less affected by nodes that are relatively isolated from the network^39^. In this study, the high correlation between characteristic path length and local efficiency may be the evidence that certain nodes have less influence in normal and sedative brain. Connective strength is the mean of all connection for a node and node strength is the sum of weights of links associated with the node. The correlation between characteristic path length and connective strength or between characteristic path length and node strength could mean that each channel’s connection had immediate connection between nodes without a roundabout. The definition of clustering coefficient may be used to yield the weighted clustering coefficient by replacing the number of triangles with the sum of triangle intensities^40^. Our findings with increased characteristic path length and reduced clustering coefficient in sedation indicate that the sedation network topology is consistently farther in a small world than baseline network^41^.

In this study, we found a decrease in network parameters in the delta band. Delta band power during sleep has been reported to increase and is known to be associated with unconsciousness^42^. In recent studies using propofol, global efficiency and local efficiency in the delta band increased in the transition from consciousness to unconsciousness, which is different from the present study^43^. Similar to our study, previous studies have shown that the power of the delta band decreases paradoxically in the nitrous oxide sedation^15,16^, unlike in the case of using other general anesthetics^44^. Nitrous oxide does not induce deep sedation due to its weak potency, but propofol can induce deep sedation and loss of consciousness. These differences may be due to differences in sedation depth.

Beta oscillation is involved with various tasks that require the sensorimotor system^45^. We have previously reported that during nitrous oxide sedation, beta band parietal-frontal interactions are decreased^46^. Similar to previous studies, we observed significant alterations in delta and beta frequency bands during nitrous oxide administration. Considering the association of these frequency bands with cognitive function, changes in the brain network dynamics of these bands may impair consciousness.

In the present study, the effect of nitrous oxide was most prominent in the alpha frequency band compared to the other frequency bands we examined. This supports our previous observation that nitrous oxide exhibits the most pronounced frontal-parietal interactions in the alpha band^38^. Alpha oscillations are suggested to have a prominent role in the functions and communications of the entire brain and body^47^. Alpha bands are related to the access of information representing environmental knowledge^48^. The changes in brain connectivity induced by nitrous oxide suggests that sedation occurs due to the influence of alpha bands, and nitrous oxide has previously been shown to affect alpha frequency-associated brain activity^46,49^. Additionally, the alteration of brain activities related to the alpha band, have been found to be associated with the actions of various anesthetics^37,50,51^. Taken together, these findings suggest that the alpha frequency band may serve as a putative mechanism or marker for sedation and anesthesia^37^.

Changes of network connective parameters in theta and beta bands were not significant in this study. Theta oscillations are most commonly related to memory processes^52^. Nitrous oxide has been reported to produce impairment of memory function^53^. However, memory alteration was not observed in this study. This may be because the sedation time is not long enough to cause memory alteration. Gamma oscillations are associated with attentive processing of information^54^. However, there was no significant difference in the gamma band when consciousness is altered. This is because this frequency band may be affected by elimination of artifacts such as EMG and ECG.

Characteristic path length can be analyzed by dividing by frequency. As mentioned, the alpha1 and alpha2 frequencies had longer charateristic path length. Each path length between two channels had a longer statistically significant sedation effect. In Fig. 3, sedation situation had a longer functional distance than the baseline situation. Nitrous oxide reduced the difference between physical and functional distance. This means that in sedation situations, all connections of brain nodes can be loosened especially at the hub of information transfer, decreasing the global brain network efficiency. Therefore, the characteristic path length may be entirely affected by nitrous oxide sedation at alpha1, alpha2 frequencies.

However, the present study has several limitations. Even though the same nitrous oxide concentration was administered to all participants, individual differences in the response to this concentration may have affected sedation depth. Although all participants maintained consciousness and responded to verbal commands during nitrous oxide sedation, some changes in mood were observed. Therefore, all participants were in a state of conscious sedation during nitrous oxide administration.

In conclusion, nitrous oxide induces changes in the brain network, decreasing the efficiency of information processing in the frequency bands important for cognitive processes. Therefore, the decrease in cognitive processes resulting from brain network changes, may be associated to the sedative mechanism of nitrous oxide.

## Material and Methods

### Volunteer recruitment

All experimental procedures were approved by the Institutional Review Board (IRB No: CRI15022) Seoul National University Dental Hospital, Seoul, Korea), and was in accordance with the declaration of Helsinki. Participants were 20 healthy volunteers between the ages of 20–40. All volunteers provided written informed consent. Physical examinations and interviews were conducted to confirm that participant had no history of cardiovascular, respiratory, renal, endocrine, hematologic, gastrointestinal, central nervous system, or psychiatric disease. Participants who had such medical disease were excluded from this study.

### Nitrous oxide sedation protocol

The nitrous oxide sedation protocol consisted of four stages. Prior to nitrous oxide administration, electroencephalogram (EEG) recordings were made for 5 min. We considered this awakening state as a baseline. Subsequently, using a facemask suited to the participant, 100% oxygen was administered at a flow rate of 6 L/min, to confirm appropriate breathing. Sequential administration of 30% and 50% nitrous oxide for 5 min each, respectively, was performed. After nitrous oxide administration, 100% oxygen was administered for at least 5 min until volunteers reached consciousness level before administering nitrous oxide. Participants were instructed to fast for 8 h before the experiment, and to keep their eyes closed and relaxed during the EEG recordings. During the experiment, we verified the state of consciousness and sedation by responses to verbal commands.

### EEG signal acquisition

Continuous EEG recordings were obtained from all participants (sampling rate = 2048 Hz, low passed with 417-Hz cutoff frequency). EEG data were acquired with custom-made software (Biosemi, https://www.biosemi.com/), and 32 electrodes were placed according to standard 10–20 International placement (Fp1, AF3, F7, F3, FC1, FC5, T7, C3, CP1, CP5, P7, P3, Pz, PO3, O1, Oz, O2, PO4, P4, P8, CP6, CP2, C4, T8, FC6, FC2, F4, F8, AF4, Fp2, Fz, Cz). Data were saved and analyzed offline. We extracted EEG epochs for three minutes until 10 seconds before the sedation was started and three minutes before 100% oxygen was administrated, respectively. We considered these EEG epochs as baseline and sedation states, respectively. Data were down-sampled at 128 Hz with a 60-Hz notch filter. The Laplacian methods was used for spatial filtering^55^. All data were manually inspected by the researchers to exclude artifacts such as electromyogram (EMG) or electrocardiogram (ECG). Average Fourier cross-spectral matrices were computed for frequency bands including, delta (2–3.5 Hz), theta (4–7.5 Hz), alpha1 (8–9.5 Hz), alpha2 (10–12.5 Hz), beta1 (13–18 Hz), beta2 (18.5–21 Hz), beta3 (21.5–30 Hz), and gamma (30.5–44 Hz).

### 1/f dynamics

The power spectrum (PS) of EEG is biological time series. It often tends to have an inverse relationship between the amplitude of power and the frequency. This inverse relationship can be expressed to a function of frequency (f) as follows^56^:

1 $${\rm{PS}}({\rm{f}})={\rm{\psi }}\,\ast \,{{\rm{f}}}^{-{\rm{\alpha }}}({\rm{with}}\,{\rm{\psi }}\,{\rm{real}})$$

In formula (1), by log transformation between both side, the α represents the rate at which the power spectrum decreases at log(PS(f)) as follows:

2 $$\log ({\rm{PS}}({\rm{f}}))=-\,{\rm{\alpha }}\ast \,\log ({\rm{f}})+\,\log ({\rm{\psi }})$$

In formula (2), the α could provide an estimate of the linear correlation length within the time series. This indicates that α, the slope of the power spectrum, may be able to provide an index of “temporal memory effects” in the time series^57^. That may explain the characteristics of two extreme noises: white noise and Brownian noise. White noise does not correlate to time and therefore, is not related to frequency bands. The lack of correlation causes the flat power spectrum of white noise. Brownian noise (or random walk noise), displays correlations over time indicating that, in a “random walk” pattern, the position of a particle at time t + 1, depends on its position at time t. The power spectra of white noise and Brownian noise are flat and proportional to f^−2^, respectively. It has been reported that the power spectrum of spontaneous neural signals may follow the general rule f^−α^, with α close to 1^21,57,58^. The relation of f^−1^ was called to “Pink noise” between white noise and Brownian noise.

The exponent α can be obtained from a linear regression between log(PS) and frequency f as follows:

3 $${\rm{Y}}=-\,{\rm{\alpha }}\ast {\rm{X}}+{\rm{\beta }}\,({\rm{Y}}=\,\log ({\rm{PS}}({\rm{f}})),\,{\rm{X}}=\,\log ({\rm{f}}),\,{\rm{\beta }}=\,\log ({\rm{\psi }}))$$

In formula (3), we calculated α for each artifact-free epoch for frequencies in the range between f = 0.0056 Hz and f = 1 Hz. The mean α was calculated for all individual epochs during the baseline and nitrous oxide sedation states. The slope for the two groups in all regions of 32 sensors was calculated by the regression analysis using ‘polyfit’ function in matlab and compared by paired Student t-test.

### Graph theory analysis

To investigate changes in brain network during nitrous oxide sedation, we calculated network parameters from graph theory analysis with undirected and weighted network. In this study, node meant the sensors of 32 electrodes. The weights meant lagged phase coherence which is the functional connectivity strength between the pairs of sensors as explained above. Node strength, functional distance, characteristic path length, clustering coefficient, and local efficiency were calculated from the baseline and sedation functional connectivity matrices, based on sensors from the Brain Connectivity Tool Box (BCT) (Version 2017-15-01)^32^.

Node strength is defined as the sum of the weight of all connections between the target node and remaining nodes in the network. Therefore, node strength indicates the strength of the individual nodes in the network. Functional distance is defined as the length of the shortest path between a pair of nodes. The functional distance matrix was computed from the connection-length matrix using Dijkstra’s algorithm^59^. Characteristic path length is defined as the average shortest path length of the network, which is the mean functional distance matrix in which the distance between two nodes is not infinity. The characteristic path length serves as a measure of global connectivity. The clustering coefficient measures the degree of local connectivity between each node and its neighbors and is calculated by estimating the number of triangles around a node. Clustering coefficient is used as a measure of local connectivity^4^. Local efficiency is a parameter that characterizes the efficiency of information transfer between the neighbors of a particular node. Local efficiency is obtained by calculating the average inverse shortest path length for the subnetwork formed by node neighborhoods.

### Statistical analysis

A Wilcoxon signed-rank test was used to detect differences between the baseline and sedative state for each network connectivity measurement and slope steepness. A Mann-Whitney U test was used to detect any potential gender differences in network parameters. Pearson correlation analysis was used to investigate the relationship between the functional connectivity value for each pair-wise combination of sensors and physical distance. The physical distance between sensors was calculated using Euclidean distance using each sensor’s x, y, and z coordinates obtained by the sLORETA program. (http://www.uzh.ch/keyinst/loreta.htm)^60^. P values less than 0.05 were considered statistically significant.
